# The Effect of Cyclic Heat Treatment on the Microstructure and Mechanical Properties of 18CrNiMo7-6 Gear Steel

**DOI:** 10.3390/ma17235855

**Published:** 2024-11-29

**Authors:** Xin Liu, Wenchao Yu, Hanlin Che, Jugan Zhang, Jiahao Zhu, Qingwei Jiang, Chi Zhang, Maoqiu Wang

**Affiliations:** 1 School of Materials Science and Engineering, Kunming University of Science and Technology, Kunming 650093, China; liuxin_lacey@163.com; 2 Central Iron & Steel Research Institute Company Limited, Beijing 100081, China; yuwenchao@nercast.com (W.Y.); jiahao_zhu0807@163.com (J.Z.); 3 Laboratory of Advanced Materials of Ministry of Education, School of Materials Science and Engineering, Tsinghua University, Beijing 100084, China; 15313133853@163.com (H.C.); 15810366786@163.com (J.Z.)

**Keywords:** 18CrNiMo7-6 gear steel, cyclic heat treatment, microstructure evolution, strengthening mechanisms

## Abstract

To avoid grain coarsening resulting from high-temperature carburizing, the effects of cyclic quenching and tempering on the microstructure and mechanical properties of 18CrNiMo7-6 gear steel were investigated. Three groups of samples were compared, which went through 0/1/3 times of quenching–tempering cycles after initial pseudo-carburizing. The variations in grain size, hardness, tensile strength, and toughness were systematically assessed using a series of experimental techniques. The experimental results indicate that the austenite grain size decreases from 14.8 μm to 5.0 μm as the number of cycles increases, accompanied by improved grain uniformity, which is beneficial to fine-grain strengthening mechanisms. During the phase transition, defects in the original martensite structure are transferred to the newly formed austenite, with the energy stored during the martensitic-to-austenitic transformation driving the grain refinement process. However, after several cycles of quenching and tempering, the release of some residual stresses and dislocations reduces the driving force for recrystallization, limiting further grain refinement. Although the strength decreased slightly after three cycles due to a reduction in dislocation density, toughness increased to a maximum of 172 J/cm^2^, primarily due to the enhancement of grain refinement and grain boundary density, which effectively hindered crack propagation. This study confirms the efficacy of cyclic heat treatment in refining grain structure and improving both strength and toughness, thereby contributing valuable insights to the research and development of high-performance gear steels.

## 1. Introduction

As a core component of transmission systems, gears are extensively used in various mechanical engineering fields, including transportation, energy generation, and lifting operations. 18CrNiMo7-6 steel, known for its high strength, wear resistance, toughness, and hardenability, is an ideal material for critical components such as gears and bearings [1,2,3,4]. In practical applications, gears endure multiple and complex stresses, which necessitates specific material properties; the gear surface must be sufficiently hard to maintain high strength and wear resistance under heavy loads, while the core must exhibit good toughness to withstand various stress states, including rotation, bending, and tensile compression, ensuring reliability and durability. Carburizing technology not only enhances surface hardness and wear resistance but also preserves core toughness [5,6]. However, prolonged high-temperature carburizing can lead to grain coarsening in austenite [7]. For gear steels, the typical requirement for austenite grain size is within grade 5–8, with even stricter demands for high-performance gear steels [8]. Therefore, controlling the austenite grain size in carburized scenarios has become a critical challenge [9,10]. Recently, microalloying have been increasingly employed in the development of new gear steels [11,12,13,14,15]. For example, the addition of elements like Nb can precipitate a second phase at high temperatures, effectively pinning grain boundaries and inhibiting austenite grain growth [16,17]. However, due to the small amount of microalloying elements, the effect on grain refinement is limited, making the optimization of heat treatment processes a more effective strategy for achieving grain refinement.

Grain refinement is widely recognized as an effective method to enhance strength and toughness. Cyclic heat treatment achieves grain refinement by inducing phase transformations through repeated heating and cooling. Unlike other fine-grain strengthening methods, such as microalloying, cyclic heat treatment requires no additional alloying elements, plastic deformation, or alteration of the material’s composition and shape, allowing for grain refinement to the micron scale [18]. Quenching and tempering are common heat treatment processes; quenching improves steel’s strength and toughness, while tempering reduces the formation of microcracks during phase transitions [19]. Wang et al. [20] applied cyclic quenching with oil cooling at 1000 °C to 45 # steel (a medium-carbon steel commonly used in mechanical applications) and found that after 10 cycles, the original austenite grain size decreased from 11.3 μm to 1.9 μm, increasing tensile strength to 1700 MPa and elongation to over 5% while also refining the carbide. Similarly, Wang et al. [21] used cyclic quenching with water cooling at 810 °C on 18CrNiMo7-6 steel, observing significant austenite grain refinement from 14 μm to 3.4 μm after three cycles. Further cycling beyond three times did not lead to additional refinement, with hardness remaining constant and ultimate tensile strength (*σ* = 1397 MPa) and total elongation (TE = 9.69%) peaking after three cycles. Pan et al. [22] employed cyclic quenching on Nb-Mo micro-alloyed medium manganese steel and noted a gradual transformation in austenite morphology from a combination of lamellar and equiaxed grains to predominantly lamellar grains. Liu et al. [23] observed that the tensile strength of medium manganese steel attained 838 MPa following two quenching cycles, significantly enhancing the stability of residual austenite. Furthermore, Cui et al. [24] reported a decrease in austenite grain size from 60.16 μm to 15.06 μm, with a tensile strength of 2834 MPa after the cyclic quenching of AISI M50 steel. The effects of cyclic quenching and tempering on the microstructure and mechanical properties of gear steel are still inadequately explored. Thus, investigating how cyclic heat treatment influences these properties is crucial.

This study investigates the impact of cyclic heat treatment on the microstructure and mechanical characteristics of gear steel. To prevent excessive residual stress after cyclic quenching, which may result in microcracks and degrade mechanical integrity, the heat treatment process used in this study consists of two steps, namely quenching and tempering. 18CrNiMo7-6 gear steel was chosen as the material for the investigation, and a pseudo-carburizing process was employed to simulate the microstructural changes during carburizing. Different quenching and tempering cycles were then applied to evaluate their effects on the steel’s microstructure and mechanical properties.

## 2. Experimental Methods

Table 1 presents the chemical composition of the experimental 18CrNiMo7-6 steel. This steel is modified by adding Nb to enhance the pinning effect of the precipitated phase at the grain boundary. As a result, the carburizing temperature can be increased and the carburizing cycle effectively shortened. The critical temperatures Ac_1_ and Ac_3_, calculated using Thermo-Calc software (2023 a), are 690 °C and 774 °C, respectively.

**Figure 1:**
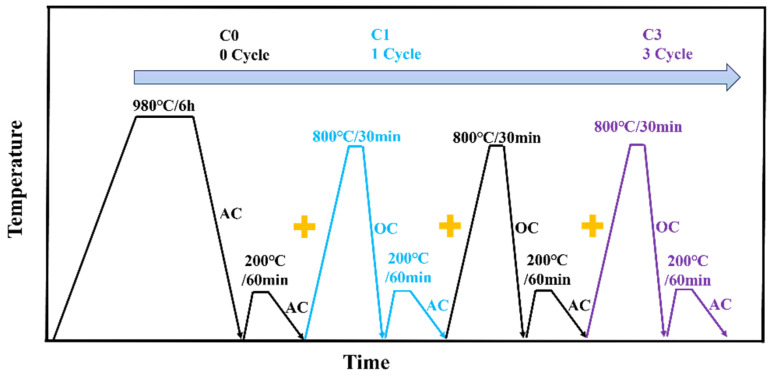
Schematic diagram of cyclic heat treatment process. (AC, air cooling; cooling rate, 18 °C/s; OQ, oil quenching).

In this study, metallographic samples (10 × 10 × 150 mm), tensile samples (Φ 6 × 65 mm), and U-notch impact samples (10 × 10 × 55 mm) were first placed in a heat treatment furnace, heated to 980 °C, and held for 6 h before air cooling. The samples were then tempered at 200 °C for 1 h (labeled C0). One set of metallographic samples (10 × 10 × 150 mm) was cut into six smaller cubes (10 × 10 × 10 mm). These samples were divided into three groups, with two samples in each group, and subjected to 1 and 3 quenching + tempering cycles (labeled C1 and C3, respectively, as shown in Figure 1). Each quenching + tempering cycle consisted of oil quenching after holding at 800 °C for 30 min, followed by tempering at 200 °C for 1 h and air cooling to room temperature.

The microstructure and grain dimensions of the specimens were examined using a scanning electron microscope (Zeiss Merlin Compact, SEM, Jena, Thüringen, Germany) operating at an accelerating voltage of 15 kV alongside an optical microscope (LEICA DMI8, OM, Wetzlar, Hessen, Germany). The specimens underwent mechanical grinding, polishing, and etching with a 4% nitric acid–alcohol solution for 15s to reveal their microstructure. Austenite grains were treated with a saturated picric acid solution containing a minor amount of activator (3 drops of dishwashing liquid) at 55 °C for 20 min. The average size of the austenite grains was evaluated using the intercept method in accordance with ASTM E112-3 [25]. Metallographic specimens were polished electrolytically in a 5 vol.% perchloric acid–ethanol solution at 15 V for 50 s. The crystal orientation and grain boundary distribution of the experimental steel were analyzed via electron backscatter diffraction (EBSD) employing a TESCAN S9000X system (Brno, Czech Republic). The phase, lattice parameters, and other related characteristics were determined through X-ray diffraction (XRD) with a D8 ADVANCE system (Co target, tube voltage 35 kV, scanning speed 2°/min, Karlsruhe, Baden-Württemberg, Germany). The distribution, morphology, and dimensions of martensitic laths were examined using transmission electron microscopy (TEM, FEI Tecnai G2 F20, Hillsboro, OR, USA). In this research, all sample areas for microstructural analysis were sourced from the central region of the specimen.

For mechanical properties testing, an XHWT-1000Z micro-Vickers hardness tester (Beijing Shidaihaide Company, Beijing, China) was used with a 9.8 N load for 10 s. Five measurement points were randomly selected on the surface of each specimen, and the average value was reported as the Vickers hardness. Tensile tests were conducted using a WE-300 hydraulic tensile testing machine (Jinan, China) in accordance with the GB/T 228.1-2010 standard [26], with a strain rate of 0.00025 s^−1^. Two parallel specimens were tested, and their average values were documented as indicators of strength and ductility. Additionally, room-temperature impact tests were performed on a JBN-300B impact tester following the GB/T 229-2007 standard [27]. Again, two parallel specimens were tested, and their average value was reported as a measure of the material’s toughness. In this study, both physical and chemical phase analysis methods were employed to qualitatively and quantitatively assess the precipitated phases in the experimental steel. The electrolytic conditions were specified as follows: 3% (*V*/*V*) HCl, 5% (*V*/*V*) glycerol, and 2.5 g/L citric acid in a methanol solution, with a current density of I = 0.08 A/cm^2^ and a temperature of T = −10 °C. A qualitative analysis of the precipitated phase residue was conducted using a Bruker D8 X-ray diffractometer (Karlsruhe, Baden-Württemberg, Germany), while a quantitative analysis was performed using inductively coupled plasma atomic emission spectrometry (ICP-AES). The particle size of the precipitated phase residue was evaluated using a 3014 X-ray diffractometer (Rigaku, Tokyo, Japan) equipped with a Kratky small-angle diffraction system. Each phase analysis was conducted on samples measuring 20 mm × 80 mm × 3 mm, with five specimens being required for each analytical process.

## 3. Results

### 3.1. The Effect of the Number of Cyclic Heat Treatment Processes on the Microstructure

**Figure 2:**
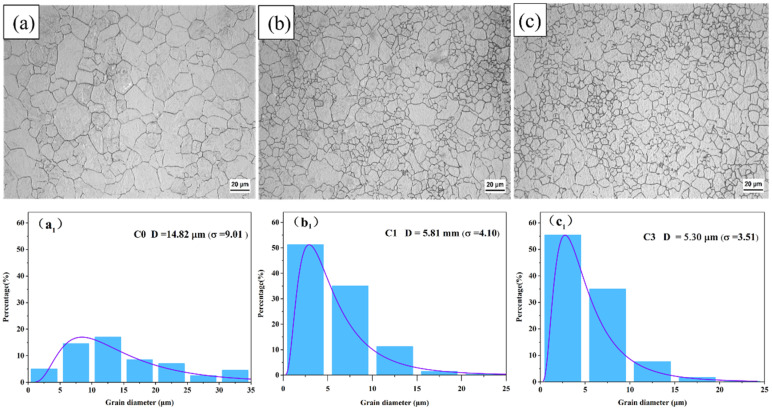
Micrographs showing the microstructures and prior austenite grains of the tested steels under different cycles. (**a**,**a_1_**) C0; (**b**,**b_1_**) C1; (**c**,**c_1_**) C3.

The morphology of the austenite grains and the primary austenite grain size of the steel after different quenching and tempering cycles are illustrated in Figure 2. Figure 2a,a_1_ presents the initial austenite structure (C0) of the experimental steel after being heated at 980 °C for 6 h, followed by a tempering process of 1 h at 200 °C. In this condition, the austenite grains appear coarse and equiaxed, with an average dimension of about 15 μm. After one cycle (C1), the primary austenite grains were significantly refined, with the grain size reduced to as small as 5.8 μm, as shown in Figure 2b,b_1_. After three cycles (C3), the primary austenite grain size decreased further to 5.3 μm, as shown in Figure 2c,c_1_. Additionally, the grain size distribution in the original state was wide, with a standard deviation (*σ*) of nine. After one and three cycles, the standard deviations decreased to 4.1 and 3.5, respectively, as shown in Figure 2a_1_–c_1_. The grain size analysis indicates that austenite grains were effectively refined, and the uniformity of fine grains increased significantly with additional cycles, demonstrating that cyclic quenching and tempering is effective for grain refinement.

**Figure 3:**
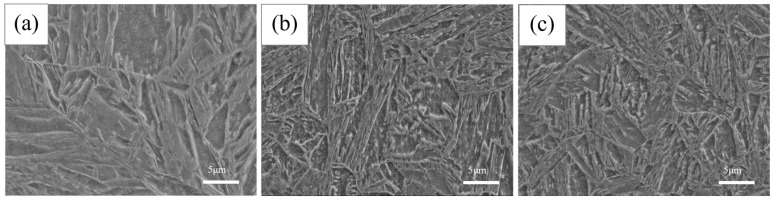
The microstructures of experimental steel subjected to cyclic heat treatment. (**a**) C0; (**b**) C1; (**c**) C3.

Figure 3 presents the microstructure in its original state and after different cycles, primarily consisting of martensite (M). It is clear that in the original state, after one cycle and after three cycles, the structure remains as lath martensite. As the number of cycles increases, no significant changes are observed.

**Figure 4:**
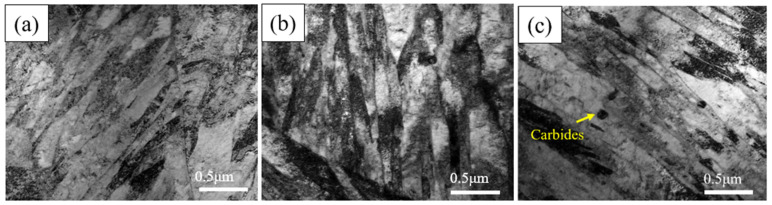
TEM micrographs of experimental steel subjected to cyclic heat treatment. (**a**) C0, (**b**) C1, and (**c**) C3 samples.

Figure 4 shows that the average width of the martensite laths decreases with an increasing number of cycles (Table 2). The average width of the C0 martensite laths is 0.15 μm, while the C1 martensite laths have an average of 0.10 μm and the C3 martensite laths are as narrow as 0.08 μm. This indicates that cyclic heat treatment can effectively refine both the prior austenite grains (PAGs) and the martensite laths. The main mechanism is that within each prior austenite grain, there are multiple packets, which are subdivided into parallel blocks. These blocks, in turn, are composed of multiple parallel martensite laths. Thus, refinement of the PAGs leads to the refinement of the martensite laths.

**Figure 5:**
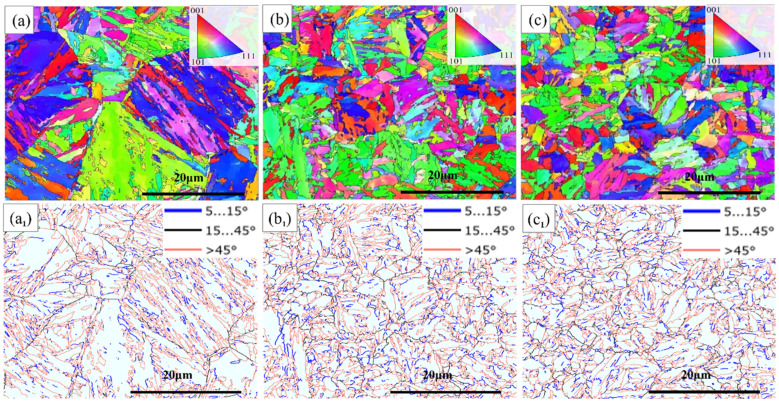
EBSD IPF + GB micrographs (**a**–**c**) and IPF color map (**a_1_**–**c_1_**) of the tested steel under different heat treatment cycles (**a_1_**–**c_1_**). C0 (**a**,**a_1_**); C1 (**b**,**b_1_**); C3 (**c**,**c_1_**); the blue lines are low-angle boundaries with misorientation angles between 5° and 15°, whereas the black and red lines indicate the high-angle boundaries with misorientation angles of 15°–45° and higher than 45°, respectively.

The EBSD IPF + GB microstructure of the experimental steel following different heat treatment cycles is illustrated in Figure 5. The blue lines signify low-angle grain boundaries (LAGBs) with misorientations ranging from 5° to 15°, while black lines represent high-angle grain boundaries (HAGBs) with misorientations between 15° and 45°. Red lines indicate HAGBs with misorientations exceeding 45°. Statistical data on the grain boundary ratio and density are provided in Table 3. The grain boundary ratio and density for samples C1 and C3 exceeded those of C0, further demonstrating that the cyclic heat treatment process effectively refines the grains. Furthermore, the grain boundary ratio and density of PAGBs in C3 surpassed those in C1, indicating that the grain refinement effect in C3 is the most significant. These findings are consistent with the previous results. The misorientation angle differences between packets and blocks typically range between 45° and 65°.

### 3.2. The Effect of the Number of Cyclic Heat Treatment Processes on Mechanical Properties

**Figure 6:**
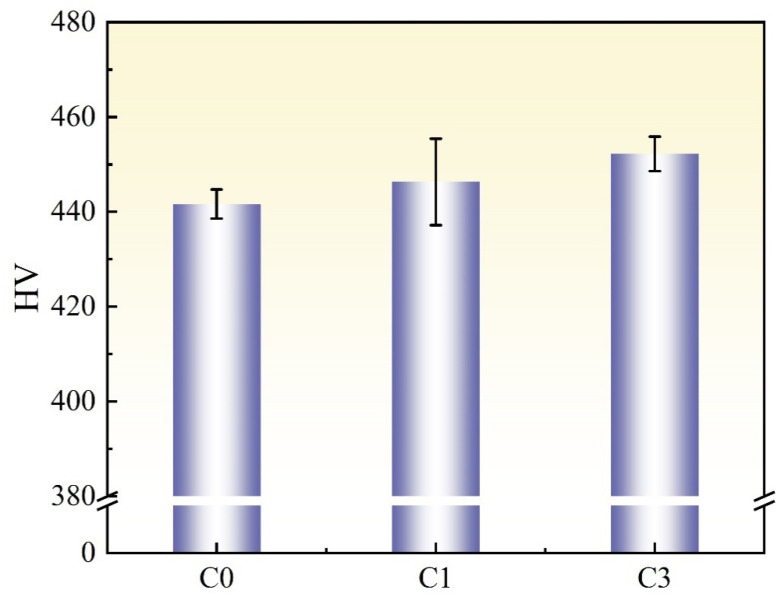
The Vickers hardness of C0, C1, and C3 samples.

To investigate the influence of cyclic heat treatment on hardness, three samples were tested using a Vickers microhardness tester (Beijing Shidaihaide Company, Beijing, China), and the results are shown in Figure 6. Before cyclic heat treatment (C0), the experimental steel exhibited a lath martensitic structure with a hardness of 441 ± 3 HV. After austenitizing at 800 °C followed by oil quenching and tempering, the hardness of sample C1 increased to 446 ± 9 HV. After three cycles (C3), the hardness of the steel further increased to 452 ± 3 HV. These results show a slight increase in hardness after cycling, but the number of cycles had a minimal impact on hardness.

**Figure 7:**
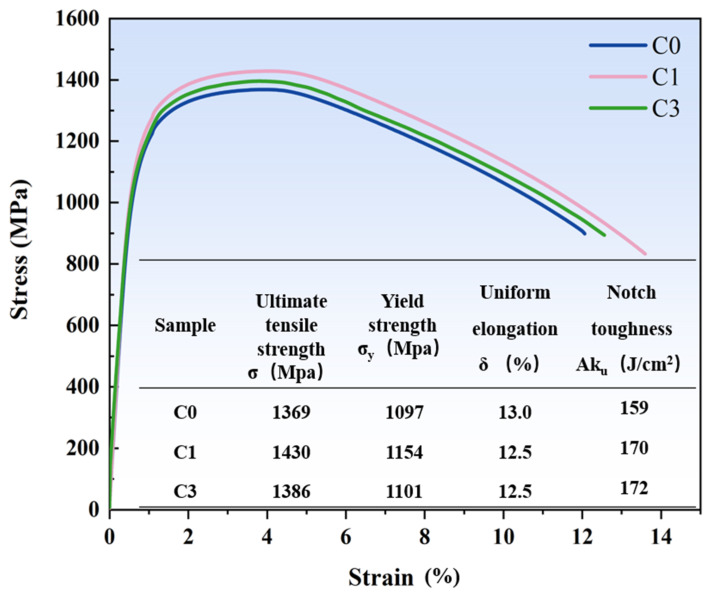
Tensile properties and impact toughness test of C0, C1, and C3 samples.

The tensile properties of the steel after different cycles are presented in Figure 7. For sample C0, the ultimate tensile strength (*σ*) was 1369 MPa, the yield strength (*σ*_y_) was 1097 MPa, and the uniform elongation (UEL) was 13%. For sample C1, *σ* increased to 1430 MPa, *σ*_y_ to 1154 MPa, and UEL was 12.5%. For sample C3, *σ* was 1386 MPa, *σ*_y_ was 1101 MPa, and UEL remained at 12.5%. Although the *σ* and (*σ*_y_) of the C3 sample were slightly lower than those of C1, both C1 and C3 exhibited higher strength than C0. According to the impact test results, the impact toughness of C0 was 159 J/cm^2^, C1 was 170 J/cm^2^, and C3 was 172 J/cm^2^, indicating that strength and toughness improved once cyclic heat treatment was applied. In summary, the strength and toughness of the samples after cyclic heat treatment were higher than in their original state, with sample C1 demonstrating the best combination of strength, ductility, and toughness.

## 4. Discussion

**Figure 8:**
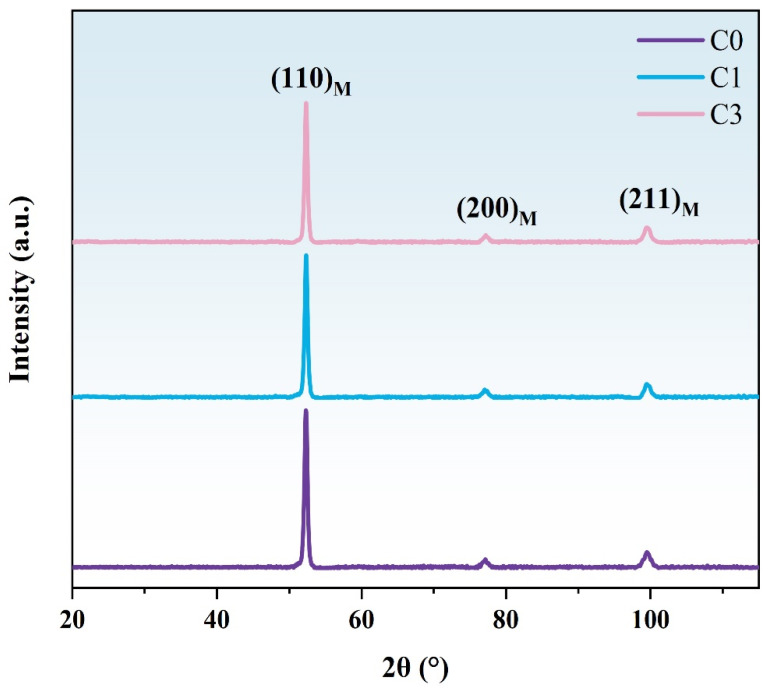
XRD patterns of experimental steel subjected to cyclic heat treatment. Purple lines are C0 samples, blue lines are C1 samples, and pink lines are C3 samples.

**Figure 9:**
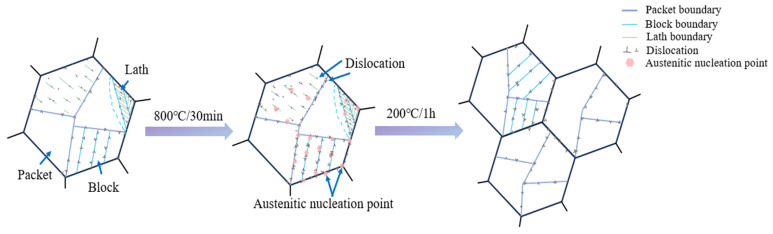
Schematic illustration of the microstructure evolution of austenite nucleation and tempering during cyclic heat treatments.

Figure 3 and Figure 4 present SEM and TEM images of the original sample (C0) air-cooled from heating at 980 °C for 6 h and tempering at 200 °C for 1 h. The microstructure is primarily composed of low-carbon lath martensite. XRD analysis was performed to examine the phases in the original state and after different cycles (Figure 8). The results indicate that both the original and cycled samples consist of martensite, with no other phases detected. After holding at 980 °C for 6 h and air cooling, a fully martensitic structure is formed due to the high hardenability of the steel and the small sample size (10 × 10 × 10 mm). Following one quenching and tempering cycle, the grain size of the C0 sample is refined to approximately 5 μm. After three cycles, the grain size remains largely unchanged, but the uniformity of grain refinement improved. This improvement is largely attributed to the transformation of new martensite, which involves a diffusional phase transition occurring in two main stages; first, martensite begins to decompose and supersaturated carbon precipitates at the lath, block, packet release, and PAGBs to form carbides. As the heating temperature increases, the carbides dissolve and austenite nucleates at the ferrite/cementite and martensite/cementite interfaces and PAGBs, growing independently during subsequent heating [28]. In low-carbon martensite, each lath martensite unit is a single crystal structure, and its substructure primarily consists of dislocations and dislocation cells. The laths are separated by LAGBs, while packets and blocks are separated by HAGBs. At dislocations and grain boundaries, atoms are arranged irregularly, resulting in high-energy sites where new austenite preferentially nucleates [29]. From a thermodynamic perspective, nucleation at these high-energy sites reduces the system’s total energy. The cyclic refinement process is schematically illustrated in Figure 9.

The austenite grain size remains largely unchanged with increasing cycle numbers because after multiple cycles, the driving force for austenite recrystallization primarily arises from phase transformation energy storage, and recrystallization occurs mainly in the single-phase austenite region, unlike nucleation growth driven by interface energy reduction. Furthermore, structural defects from the original martensite are carried into the new austenite during phase transformation. The martensite-to-austenite transformation is accompanied by volume changes and thermal stresses, similar to recrystallization induced by plastic deformation. These residual stresses resulting from the transformation facilitate austenite recrystallization. To prevent microcracks caused by excessive internal stress during multiple quenching cycles, tempering was applied after each quenching cycle, which resulted in changes in internal stress and dislocation density as the number of cycles increased.

XRD data were used to calculate the dislocation density in the samples after different cycles. The dislocation density of the C0 sample was 2.60 × 10^11^ cm^−2^. During the heating process, the steel undergoes complete austenitization, and the following air-cooling step transforms the austenite into lath martensite. This transformation induces significant dislocation formation due to lattice distortion during cooling. Although some dislocations were eliminated during the recovery after the first tempering, the overall dislocation density remains high due to the high-hardenability martensitic phase transition. After one cycle, the dislocation density increased to 3.15 × 10^11^ cm^−2^. As the quenching and tempering cycles progressed, each tempering stage reduced residual stresses and dislocations. Despite the introduction of new dislocations during quenching, tempering processes allowed for the elimination of dislocations via mechanisms such as dislocation annihilation or climb. Consequently, after three cycles, the dislocation density decreased to 2.67 × 10^11^ cm^−2^. Furthermore, cyclic heat treatment promoted carbide formation, and these precipitates pinned dislocations, contributing to dislocation strengthening.

The strengthening mechanisms after cyclic quenching and tempering were further analyzed using yield strength [30] calculation models.

(1) $$\sigma_{y}=\sigma_{0}+\sigma_{d}+\sigma_{HP}+\sigma_{SS}+\sigma_{P}$$

where *σ_d_* is the dislocation strengthening, *σ_HP_* corresponds to the grain boundary hardening, *σ_ss_* refers to the solid solution hardening, and *σ_p_* denotes the precipitation hardening. *σ*_0_ is the resistance of the lattice to dislocation slip. For a BCC matrix in iron and steel, the *σ*_0_ is usually approximately 50 MPa [31,32].

According to Kelly [33], after quenching, only a small fraction of carbon atoms in lath martensite remains in the lattice voids of the solid solution, while the majority accumulate near dislocations. When calculating solution strengthening, the segregated carbon at dislocations and the carbon content in the second phase must be excluded. The experimental steel contains approximately 0.20 wt.% carbon, and the contribution of solution strengthening is minimal; thus, it can be considered negligible. The precipitated phase in steel can effectively hinder dislocation slip [34]. In this study, the average carbide size in the experimental steel is less than 1 μm, so the classical Orowan model [35] is employed to calculate precipitation strengthening.

(2) $$\sigma_{p}=(\frac{0.538\mu b\sqrt{V_{f}}}{2r})\ln\left(\frac{r}{b}\right)$$

where *V_f_* is the volume fraction of the precipitated phase, *μ* is the material’s shear modulus, *b* is the Burgers vector, and *r* is the equivalent radius. Based on the analysis of multiple TEM images, the equivalent radii of M_3_C carbides for C0, C1, and C3 samples are 58 nm, 70 nm, and 47 nm, with corresponding volume fractions of 0.111%, 0.179%, and 0.096%, respectively. The equivalent radii of MC carbides are 6.9 nm, 7.4 nm, and 7.7 nm, with volume fractions of 0.039%, 0.051%, and 0.052%, respectively. The total contribution of M_3_C and MC carbide precipitation strengthening for C0, C1, and C3 is calculated to be 87 MPa, 99 MPa, and 101 MPa, respectively.

The main strengthening mechanisms include dislocation strengthening and grain boundary strengthening. Dislocation strengthening occurs due to the increased density of dislocations, which impede their motion on the glide plane and result in enhanced strength. The formula for calculating dislocation strengthening is as follows [36]:

(3) $$\sigma_{d}=M\alpha \mu b\sqrt{\rho }$$

where *M* is the Taylor factor, *α* is the dislocation interaction strength factor, and *ρ* represents the dislocation density. For the lath martensite matrix, *μ* = 76 GPa, *M* = 3, *α* = 0.25 [37], and $b=\frac{\sqrt{3}}{2}a$, where *a* is the lattice constant. The contribution of dislocation strengthening in the original state was calculated to be 726 MPa. After one cycle, dislocation strengthening increased to 780 MPa, but after three cycles, it decreased to 736 MPa.

Grain boundary strengthening arises from the hindering effect of grain boundaries on dislocation movement. According to the Hall–Petch relationship [38], grain refinement significantly enhances material strength. The formula is as follows:


(4) $${\sigma_{\mathrm{HP}}=k_{\mathrm{HP}}d}^{-\frac{1}{2}}$$

where *k*_HP_ is the Hall–Petch coefficient and *d* is the effective grain size. In this study, the size of the block [39] was taken as the effective grain size, and the block sizes of C0, C1, and C3 were measured as 1.06 μm, 0.99 μm, and 1.01 μm, respectively, based on EBSD images of the experimental steel. With $k_{\mathrm{HP}}=0.21\;MPa\cdot m^{1/2}$ [32], the fine-grain strengthening contribution was calculated as 203 MPa for C0, increasing to 211 MPa after one cycle, and slightly decreasing to 208 MPa after three cycles.

**Figure 10:**
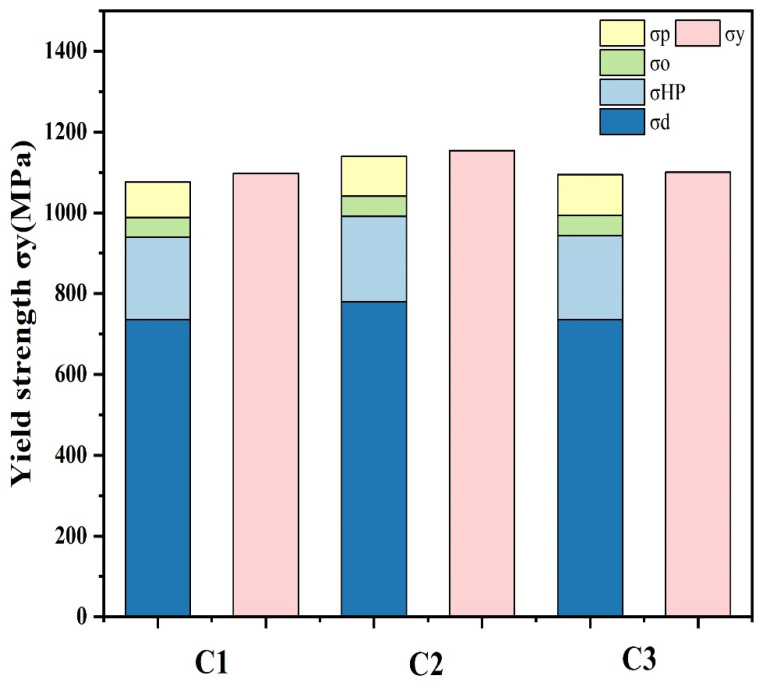
Comparison of theoretical strengths for C0, C1, and C3 samples, showing contributions from dislocation and fine-grain strengthening compared with experimental results.

By adding these above contributions, the theoretical yield strengths for C0, C1, and C3 were calculated to be 1066 MPa, 1140 MPa, and 1095 MPa, respectively, which aligns with the experimental trends shown in Figure 10. These findings indicate that cyclic heat treatment initially increases dislocation density and promotes grain refinement, leading to enhanced strength and hardness. However, after multiple cycles, dislocation density begins to decrease, suggesting that structural defects like dislocations provide energy for re-nucleation, and some dislocations are eliminated through mutual annihilation or climb mechanisms during repeated quenching and tempering, reducing the contribution of dislocation strengthening. The martensitic packet and block structures improve steel toughness, and the increased grain boundary density of HAGBs enhances crack resistance, further improving the material’s toughness [40]. Additionally, the increased density of HAGBs and LAGBs acts as a barrier to crack propagation, contributing to the material’s toughness [41].

## 5. Conclusions

In this study, the effects of cyclic quenching and tempering on gear steel were analyzed using SEM, TEM, XRD, and mechanical testing. The key findings are as follows:

1. The cyclic heat treatment significantly reduced the average austenite grain size from 14.8 μm to approximately 5.0 μm. With increasing cycle numbers, the uniformity of the refined austenite grains improved. Structural defects in the original martensite were transferred to the new austenite during phase transitions, with the phase transformation energy stored in the martensite-to-austenite process driving the cyclic refinement. However, after several cycles, the elimination of residual stresses and dislocations limited the recrystallization driving force, causing the grain size to reach a refinement limit.
2. The tensile and yield strength of the specimens increased following cyclic heat treatment, with strength peaking after one cycle (*σ*: 1430 MPa; *σ*_y_: 1154 MPa). This enhancement is primarily due to dislocation and fine-grain strengthening. However, after three cycles, the strength slightly decreased (*σ*: 1386 MPa; *σ*_y_: 1101 MPa), largely due to the reduction in dislocation density during tempering, which reduced its contribution to strength.
3. Toughness improved with increasing cycle numbers, reaching a maximum of 172 J/cm^2^ after three cycles. This improvement is attributed to grain size reduction through cyclic refinement, which increased grain boundary density and effectively impeded crack propagation. Additionally, the refinement of the packet and block structures further enhanced the material’s toughness.

## Figures and Tables

**Table 1:** The composition of the 18CrNiMo7-6 steel (wt.%).

C	Si	Mn	S	P	Cr	Ni	Mo	Nb	Fe
0.21	0.19	0.57	≤0.005	≤0.005	1.69	1.66	0.31	0.032	Bal.

**Table 2:** The average width of martensite after different heat treatment cycles.

Sample	Average Width of Martensite Lath
C0	0.15 μm
C1	0.10 μm
C3	0.08 μm

**Table 3:** Grain boundary ratio and density of tested steels measured by EBSD.

Sample	Ratio of Grain Boundary			Density of Grain Boundary [μm^−1^]		
	LAGB	HAGB		LAGB	HAGB	
	(5°–15°)	(15°–45°)	(>45°)	(5°–15°)	(15°–45°)	(>45°)
C0	15.0%	8.3%	76.7%	0.07	0.04	0.13
C1	15.8%	11.4%	72.8%	0.28	0.18	0.49
C3	16.0%	13.1%	70.8%	0.28	0.20	0.50

## Data Availability

The original contributions presented in the study are included in the article, further inquiries can be directed to the corresponding author.
